# Impact of COVID-19 on Pregnancy and Maternal-Neonatal Outcomes: A Narrative Review

**DOI:** 10.7759/cureus.31397

**Published:** 2022-11-11

**Authors:** Sweta Sahu, Guddi Laishram, Asmita Rannaware, Sonali G Choudhari

**Affiliations:** 1 Community Medicine, School of Epidemiology and Public Health, Jawaharlal Nehru Medical College, Datta Meghe Institute of Medical Sciences, Wardha, IND

**Keywords:** maternal-fetal transmission, miscarriage, vertical transmission, maternal health, gestation, pregnancy, covid-19, sars-cov-2

## Abstract

The pandemic coronavirus disease (COVID-19) has caused an unprecedented worldwide health emergency. The pandemic increased the susceptibility of pregnant women to maternal and fetal complications. Elderly and patients with comorbidities were also at high risk during the pandemic times. Further evidence supports that COVID-19 is not only a respiratory infection but possibly affects other organ systems, including the placenta. The key objective of this review is to explore the literature on COVID-19-affected pregnancies and study the pandemic's impacts on maternal, perinatal, and neonatal outcomes. We used Google Scholar and PubMed (Medline) for relevant literature searches. The clinical manifestations in pregnant women, fetus outcome, vertical transmission, and early and late pregnancy impacts are combined in database studies. Women should receive special attention for COVID even though most of the COVID-19-positive pregnant women had no symptoms or had minor ones. It was found that most pregnant women with COVID-19 had mild and few symptoms and that the effect on the fetus was insignificant. However, in some women, miscarriage and fetal growth retardation were seen as a consequence of the infection.

## Introduction and background

COVID-19 was first discovered in 2019 in Wuhan, China; two hundred-seven million people have been infected by it, and it has caused more than four million deaths worldwide [1]. The first case of COVID-19 in the United States was reported on January 22, 2020 [2]. On February 11, 2020, the WHO called the novel coronavirus SARS-CoV-2. The WHO announced this situation as an International Public Health Emergency [3]. The WHO was yet to determine the type of virus responsible for these outbreaks. There was no evidence of how the people were contracting it. It became a universal public health problem, affecting millions worldwide [4].

Many reviews and reports were published on the effects of SARS-CoV-2 on antenatal women in their first, second, and third trimesters of pregnancy. A widespread SARS-CoV-2 study of antenatal mothers in some counties revealed that, like most people, antenatal mothers infected with SARS-CoV-2 were symptomatic [1]. Most researchers focused on the effects of SARS-CoV-2 on antenatal mothers. It was difficult to classify the normal antenatal mothers from SARS-CoV-2 infected mothers because of the close physiological manifestations in pregnancy and SARS-CoV-2, such as changes in the immune system, respiratory system, cardiovascular function, and coagulation mechanism. These changes in pregnancy are physiological and hormonal. Thus it becomes difficult to distinguish whether these changes were due to SARS-CoV-2 or the pregnancy itself. Antenatal Mothers are at high risk of infection and maternal mortality; however, information on clinical symptoms of SARS-CoV-2 in pregnancy is inadequate to comment on the same. After comparing the effects of SARS-CoV-2 on pregnant and non-pregnant women, the virus's susceptibility in both groups was evaluated [5]. Some studies stated that antenatal mothers and their infants were more susceptible to infections than non-pregnant women [3,6]. One study suggests that amongst the antenatal mothers, around 10% were admitted to the hospital for the symptoms of SARS-CoV-2 [4]. The question of whether pregnant women are at increased threat of infection with SARS-CoV-2 or whether they have serious side effects is very pressing [7]. Complete testing in pregnancy of SARS-CoV-2 was advised during the pandemic times. Methods for tracing the exact spread of the virus were implemented. Data related to serological tests like D-dimer, C-reactive protein, virological tests, swab cultures, throat cultures, and neonatal tests were proposed at various stages, which defined the timely spread of SARS-CoV-2 [8]. Between October and November 2020, the WHO convened a team of multidisciplinary experts worldwide to evaluate the evidence and propose a first-phase compliance program to prevent the rapid spread of SARS-CoV-2 [9].

There is abundant information on the effects of acute maternal respiratory infection of SARS-CoV-2 on the fetus in the first or second, and third trimesters of pregnancy [10]. Postpartum SARS-CoV-2 in the third trimester and the admission of the infected mothers in the ICU also suggest that SARS-CoV-2 comprises placental pathology [11]. The virus affects the reproductive function of pregnant women. It increases the risk of fetal death or anomalies, a threat to a pregnant woman and the child after birth. There is evidence of COVID-19 Infected antenatal mothers having a miscarriage or premature birth in the second trimester. There is also a high mortality risk before or during childbirth [7,12]. According to CDC, from March 2020 to September 2021, the data shows that out of 653 births, 273 newborns were affected with SARS-CoV-2 [13]. We reviewed a study comparing before-birth and after-birth data of COVID -19 infected women [14].

## Review

This paper discusses the effect of SARS-CoV-2 and its maternal and fetal outcomes. Our report is based on previously presented domestic and worldwide literature over a search of multiple sources. We searched several websites for recent related statistics and literature in Google Scholar, Sci-Hub, PubMed, and ResearchGate. The objective was to review this article to analyze pregnant women's risk during SARS-CoV-2 and fetal effects.

According to the report by Allotey et al., on September 1, 2022, a total of 67,271 pregnant women who came or were hospitalized for any reason were suspected or confirmed for SARS-CoV-2. Around 40% of these females suffered from fever, and 41% had cough symptoms [15]. Out of 118 pregnant women, about 86%, 41%, and 14% of antenatal mothers suffered from fever, cough, dyspnea, and diarrhoea, respectively (May 20, 2022) [16]. Similar findings were obtained by Dashraath et al. (March 23, 2020), who found that fever was 84% and cough was 28% in 55 pregnant COVID-affected women. These were the most common clinical symptoms in pregnancy with SARS-CoV-2 [17]. Also, According to Chen et al. (June 18, 2020), 75% and 73% of 112 women suffered from fever and cough, respectively, as common warning symptoms [18]. The same is shown in Table 1.

**Table 1:** Shows the most common symptoms seen in antenatal mothers with COVID-19

Symptoms	Allotey et al. (%) [15]	Yu et al. (%) [16]	Dasraath et al. (%) [17]	Chen et al. (%) [18]
Fever	40%	86%	84%	75%
Cough	41%	14%	28%	73%
Dyspnea	-	14%	18%	-
Diarrhoea	-	14%	-	-

Discussion

Susceptibility, Severity, and Clinical Course

Limited evidence is available to address this issue, despite the possibility that physical, mechanical, and immunologic changes in pregnancy might affect exposure to SARS-CoV-2. The prevalence of SARS-CoV-2 infection among antenatal mothers has been documented in recurrent studies, with estimates ranging from 3-20% [19]. According to a study from Washington state, pregnant women had higher infection rates (13.9 per 1000 deliveries) when compared to non-pregnant women of age group 20 to 39 (7.3 per 1000 people) [20]. It isn't easy to separate the variations in susceptibility from various exposure, even with better incidence statistics. In conclusion, there is inadequate evidence to say whether or not pregnancy makes one more prone to SARS-CoV-2 [5].

The most delicate statistics are from the CDC's SARS-CoV-2 surveillance system, which involved over 400,000 people of the reproductive period with characteristic SARS-CoV-2 and familiar for age, race, culture, and fundamental therapeutic problems. Pregnant women had three times the risk of being admitted to an ICU (10.1 vs. 3.9 per 1000), 2.9 times the risk of needing invasive ventilation (2.9 vs. 1.1 per 1000 cases), and 1.7 times the risk of dying (1.5 vs. 1.2 per 1000 cases) [21].

Placenta and SARS-CoV-2

Several research papers have been published on vertical transmission of SARS-CoV-2 through the placenta (the presence of SARS-CoV-2 in the placenta was more recurrent in women suffering from preeclampsia or pregnancy-included hypertension. It was also found that additional severe hypertensive complaints were related to rising viral infection) [22]. The face of SARS-CoV-2 has been perceived in mid-trimester placenta samples. However, whether the virus was present due to a severe infection or aided by injuries caused by other conditions is unknown. After the automatic death of the fetus at 19 weeks of pregnancy, SARS-CoV-2 was obtained from reverse transcription-polymerase chain reaction (RT-PCR) swabs [23]. SARS-CoV-2 was similarly more pronounced in placenta and navel samples after a miscarriage at 22 weeks gestation [12]. However, SARS-CoV-2 was not noted in amnionic liquid in the case of six infected mothers. The placenta and mucous membranes of the vagina were examined and found to be free of defects. The test results for viral amniotic fluid in an infected woman's placenta and cervical blood do not show the viral infection. Still, virus manifestations have been detected in fetal tissues analyzed in these cases [14]. There was a notable difference in weight and volume of the placenta of 29 SARS-CoV-2 infected mothers (out of 322 placental samples) [24].

Direct Transmission of SARS-CoV-2

SARS-CoV-2 is spread from person to person contact through respiratory drops, intimate relationships, and sprays (Chan et al., 2020) [25]. Recent findings of the immediate spreading of SARS-CoV-2 infected antenatal mothers to newborn children pose an additional risk to newborns. The placenta is the line of vertical transmission of the infection from mother to child. [26]. We chose to examine 38 papers investigating the relationship between COVID-19 and pregnancy. These studies used samples of newborns who SARS-CoV-2-infected mothers delivered. The claim of direct vertical transmission through antenatal infection with SARS-CoV-2 was not yet clear [7].

Maternal Health and the Effects on Infants

**Figure 1:**
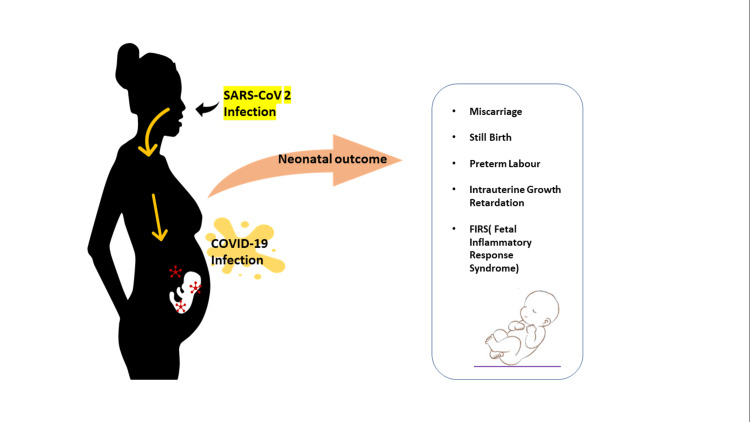
Neonatal outcomes due to SARS-CoV-2 in pregnancy Image credit: Author Sweta Sahu

There is a limitation of records on the effects of maternal infection throughout the first trimester or second trimester of pregnancy. In mothers with SARS, the chance of a miscarriage or stillbirth is more than 25%. In the recent SARS-CoV-2 epidemic, a systematic study conducted on two hundred ninety-five pregnant women found a risk of abortion/miscarriage of 1.4% [6]. Fetal problems for SARS-CoV-2 include preterm birth (39%), intrauterine growth retardation (IUGR; 10%), and stillbirth (2%). Fever, with a temperature of 38.1−39 degrees Celcius, was a recurrent symptom of SARS-CoV-2. A group of studies on antenatal mothers with other diseases did not show a rising risk of genetic disabilities from maternal sepsis in the first trimester [17]. A Wuhan, China, children's hospital study (March 6, 2020; Zeng et al.) informed that three of 33 childbirths to SARS-CoV-2-infected mothers needed neonatal ICU (NICU) intervention due to dyspnea, fever, and fatigue [27]. In some studies, even when newborns were verified for SARS-CoV-2, they had no specific symptoms or indications [16]. Neonatal outcomes due to SARS-CoV-2 in pregnancy are shown in Figure 1.

Childbirth and the Health of the Newborn

A quick response time of investigation was essential to facilitate the child's birth by a mother infected with SARS-CoV-2. Once resources are accessible, all mothers are confirmed for SARS-CoV-2 during childbirth, irrespective of their medical status, to avoid the nosocomial spread in some patients and health workers [28]. Premature birth and delivery appear more common in antenatal women with SARS-CoV-2, with further possibilities of newborns being admitted to the NICU due to other complications [15]. Antenatal mothers infected with SARS-CoV-2 were shifted to a particular room to facilitate the rehabilitation of the mothers and newborns in case of emergencies.

SARS-CoV-2 Contributes to Early Pregnancy

There is insufficient data on the first-trimester gestational period in SARS-CoV-2-infected mothers. There is little evidence of the effect of COVID on maternofoetal outcomes in the first trimester of pregnancy (during 12 weeks of pregnancy). Recurrent flu has been linked to an increased chance of miscarriage, and it will be necessary to look at demographics and community tests to determine if this is true of SARS-CoV-2 [29].

SARS-CoV-2 and Late Pregnancy

We may exclude the effect of other infections in late pregnancy (over 24 weeks of pregnancy); SARS-CoV-2 might raise the chance of unfavourable outcomes such as contraception, premature birth, and stillbirth [29]. As of January 2020, a series of case studies and group studies describing the introduction of SARS-CoV-2 and pregnancy-related research papers have been published. Few studies suggest a high risk of infection from SARS-CoV-2 throughout pregnancy. A total of 31 articles were received, and 12,260 women reported the results of antenatal mothers through SARS-CoV-2 certificates and their infants [30].

COVID-19 Vaccination in Pregnancy and While Lactating

On January 29, 2021, the WHO CoV-2 review recommended that antenatal mothers are a vulnerable group to the COVID-19 virus and hence should be immunized [31]. Vaccination is the most excellent option for pregnant and nursing mothers to save themselves and their newborns. Still, widely prescribed studies are needed before implementing safe vaccination protocols [32]. The US Nutrition and Medical Management recently accepted two vaccines. The Advisory Committee on Immunization Practices (ACIP) of the CDC and the American College of Obstetricians and Gynaecologists (ACOG) have suggested their use in antenatal and postnatal mothers [13].

Studies show that the injection of the mRNA vaccines causes a strong parental autoimmune reaction, which is essential for vaccine effectiveness. There is no goal to anticipate variances, even though the immune reaction to vaccination in pregnant women has not been thoroughly associated with the reaction in women who are not pregnant. Additionally, immunoglobulin G antibodies cross the placental barriers in pregnant women. [33-35]. Few books have reported widespread symptoms such as shortness of breath, shock, tachycardia, sepsis, thrombocytopenia, and sometimes even infant mortality. Other symptoms include fever, diarrhoea, vomiting, and constipation. There is a risk of gastric ulcer and sepsis in newborns [28]. The SARS-CoV-2 vaccinations have raised several myths and misconceptions, and doctors must be ready to address patient queries. There are many disbeliefs that COVID vaccination affects pregnancy. Women should feel comforted that there is no proof that COVID-19 vaccines impair fertility. [36]. The patients should also be informed that none of these vaccines can induce SARS-CoV-2 because none contain the infection, vaccines do not interrelate or change genetic material, and vaccines do not have any objectionable ingredients. The CDC advises doctors to empathize with patients' worries about receiving the SARS-CoV-2 vaccines [37].

N95 Respirators in Pregnancy (Prevention Method)

During the initial wave of the COVID-19 pandemic, changes to maternity services were made with the aim to reduce the risk of infection among both pregnant women and healthcare staff [6].

The CDC states that health workers at high risk for patients with warning signs or SARS-CoV-2 certificates use N95 masks (commonly known as FFP2 masks). However, these filters are related to airflow resistance and an increase in the volume of the dead area; they can affect maternal respiratory function and the baby's health if worn for a long time [31].

Breastfeeding

The WHO scientific summary of breastfeeding and SARS-CoV-2 released on June 23, 2020, found that 43 of the 46 women with SARS-CoV-2 breast milk samples verified for the presence of COVID virus were found negative. Only one child was infected in the remaining three out of 46 women because of breast milk consumption. Or the mode of infection (breast milk or close contact) had not been determined [38].

## Conclusions

Evidence of this unique infection changes daily, but it will take months to conclude. We know the natural effect it will have on maternal and child health. In the meantime, our priority is to confirm that all women give birth in healthy conditions and that both mother and baby are healthy. There has been insufficient data to reach impartial conclusions about SARS-CoV-2 severity or certain complications in pregnancies, as well as specific diseases and complications of birth and infertility, despite the rising number of findings and study papers available on pregnancy with SARS-CoV-2. This study contains only examples of lessons or a series of low-level evidence. SARS is related to an increased frequency of miscarriage in pregnant women. There is no confirmation that SARS-CoV-2 reasons for first-trimester spontaneous abortion. SARS-CoV-2 has also been informed to be absent from amniotic fluid, umbilical cord blood, newborn swabs culture in the throat, and breast milk.

With the worst and most common placental changes reported in SARS pregnancies and those still affected by immaturity after SARS infection, testing for pediatric stress in antenatal mothers with SARS-CoV-2 would make sense. We need to be aware of people who may be at risk during that same period, both patients and associated, to ensure that they have adequate support through these unexpected times. Finally, we need to consider our future decisions.
